# A Streptomyces tendae Specialized Metabolite Inhibits Quorum Sensing in Group A Streptococcus

**DOI:** 10.1128/spectrum.05279-22

**Published:** 2023-06-07

**Authors:** Vanessa M. Nepomuceno, Kaitlyn M. Tylor, Skylar Carlson, Michael J. Federle, Brian T. Murphy, Tiara Perez Morales

**Affiliations:** a Biological Sciences Department, Benedictine University, Lisle, Illinois, USA; b Department of Microbiology and Immunology, University of Illinois at Chicago, Chicago, Illinois, USA; c Department of Pharmaceutical Sciences, University of Illinois at Chicago, Chicago, Illinois, USA; d Department of Chemistry, University of the Pacific, Stockton, California, USA; The Ohio State University Division of Biosciences

**Keywords:** quorum sensing, *Actinobacteria*, *Streptococcus*

## Abstract

Quorum sensing (QS) is a means of bacterial communication accomplished by microbe-produced signals and sensory systems. QS systems regulate important population-wide behaviors in bacteria, including secondary metabolite production, swarming motility, and bioluminescence. The human pathogen Streptococcus pyogenes (group A Streptococcus [GAS]) utilizes Rgg-SHP QS systems to regulate biofilm formation, protease production, and activation of cryptic competence pathways. Given their reliance on small-molecule signals, QS systems are attractive targets for small-molecule modulators that would then affect gene expression. In this study, a high-throughput luciferase assay was employed to screen an *Actinobacteria*-derived secondary metabolite (SM) fraction library to identify small molecule inhibitors of Rgg regulation. A metabolite produced by Streptomyces tendae D051 was found to be a general inhibitor of GAS Rgg-mediated QS. Herein, we describe the biological activity of this metabolite as a QS inhibitor.

**IMPORTANCE** Streptococcus pyogenes, a human pathogen known for causing infections such as pharyngitis and necrotizing fasciitis, uses quorum sensing (QS) to regulate social responses in its environment. Previous studies have focused on disrupting QS as a means to control specific bacterial signaling outcomes. In this work, we identified and described the activity of a naturally derived S. pyogenes QS inhibitor. This study demonstrates that the inhibitor affects three separate but similar QS signaling pathways.

## INTRODUCTION

Bacteria are capable of intercellular communication using diffusible signals and receptors. This process, commonly referred to as quorum sensing (QS), allows for the coordination of gene expression and regulation of social behaviors. QS systems may be composed of two-component systems (TCS) and/or signal transport systems. In TCS, membrane receptors interact with their cognate signal extracellularly and lead to intracellular signal transduction. In signal transport systems, membrane transporters import the signal where it can engage cytoplasmic receptors that modulate cellular responses. This work focuses specifically on signal transport QS systems present in the human pathogen Streptococcus pyogenes (group A Streptococcus [GAS]).

The Rgg (regulator of glucosyl transferase gene) QS family is of great interest due to its diversity in bacterial cell responses (1). Receptors of this family are transcriptional regulators that contain an N-terminal DNA binding domain and a C-terminal-repeat ligand-binding domain (2–5). The signal, a peptide whose biosynthesis is ribosomally produced, has various names, such as SHP, XIP, and SIP (Short Hydrophobic Peptide, ComX Inducing Peptide, and SpeB Inducing Peptide, respectively) (5–9). For most, inactive propeptide signals are translocated across the membrane by the PptAB transporter and matured into an active form (SHP/XIP) by membrane-associated proteases (9, 10). Once outside the cell, peptide signals can be sensed by neighboring bacteria only after they are imported through Opp (oligopeptide permease) and by direct interaction with the transcriptional regulator Rgg. Rgg-peptide complexes undergo conformational changes that alter the transcriptional activity of the Rgg and hence the differential expression of gene targets.

S. pyogenes contains three Rgg QS systems with unique social responses. The RopB (Rgg1) QS system is induced in the presence of its target signal SIP or during changes in environmental pH (9, 11). Its main function is the production of the protease SpeB (12). The Rgg2/3 QS system is uniquely controlled via Rgg2 and Rgg3 (6, 13–15). The system responds to peptides SHP2/3, as well as changes in metals or carbohydrate concentrations. Activation of this QS system leads to biofilm formation, lysozyme resistance, and suppression of macrophage activation (16–18). Finally, ComR (Rgg4) responds to its peptide XIP and leads to the induction of genes required for the cryptic natural competence pathway (8, 19, 20). Further work has shown that Rgg/SHP QS systems can be externally manipulated using small molecules.

Previous work has shown that cyclosporine (CsA) and its analog valspodar can negatively regulate Rgg2/3 QS in S. pyogenes via direct interactions with Rgg2 and Rgg3 (21, 22). Inhibition by these molecules leads to a decrease in biofilm formation. CsA is an example of a molecule that can target specific Rgg molecules within S. pyogenes. A second molecule, P516-0475, was found as an agonist of the Rgg2/3 system. P516-0475 inhibits the SHP-endopeptidase PepO and leads to positive modulation of lysozyme resistance (23). Given that small molecules can modulate these Rgg QS systems, they can serve as therapeutic targets in this important pathogenic organism.

Natural products have been a significant source of therapeutics. Of those, a substantial number of antibiotic therapies are formulated products of *Actinomycetota* secondary metabolism (24). *Actinomycetota* is a phylum of Gram-positive, high G + C bacteria that inhabit a wide range of habitats and exist as terrestrial and aquatic soil dwellers, human and plant pathogens, and symbionts. In addition to possessing various morphologies, physiologies, and ecological roles, *Actinomycetota* have distinguished metabolite production abilities and dedicate a significant portion of their genome to secondary metabolism (25). The organisms in this phylum have made critical contributions to medicine, biotechnology, and ecology (26).

Secondary metabolites (SMs) are thought to serve as environmental signals that potentially regulate inter- and intraspecies interactions. Previous studies have shown that subinhibitory concentrations of SM-like antibiotics positively or negatively regulate gene expression and mediate biological functions. SMs have been documented to control a broad range of biological functions such as biofilm production, enzyme production, activation of antibiotic resistance genes, and biosynthesis of other SMs (27–30).

Thus, we sought to identify *Actinobacteria*-derived SMs that regulate Rgg-based QS systems to better understand the QS process. In this article, we focus on the identification of microbial SM **1** from a Streptomyces tendae strain (D051), which acts as a general inhibitor of the three Rgg QS pathways in S. pyogenes.

## RESULTS

### Secondary metabolite 1 acts as an inhibitor of Rgg2/3 quorum sensing.

Using S. pyogenes as a model system, our goal was to identify natural product inhibitors of Rgg QS pathways. A library of 2,500 actinomycete extracts was selected to search for inhibitors of at least one Rgg QS pathway in S. pyogenes. Our initial target was the well-described Rgg2/3 QS system (6, 14).

The screen used bacterial cultures containing transcriptional luciferase reporters. The test strain contained the promoter for the Rgg2/3 QS peptide *shp3* (*P_shp3_-luxAB*) (JCC181) (6). Exponentially growing cultures were incubated with 5 μg/mL of SM extracts followed by the addition of 200 nM SHP3. Approximately 189 out of 2,500 extracts showed 90% inhibition of luciferase activity (Fig. S1 in the supplemental material). The follow-up screen tested the extracts using a control strain that contained the constitutive promoter for *recA* (*P_recA_-luxAB*) (JF02) to eliminate any extracts that may affect luciferase activity or transcription in general. We also determined if the remaining extracts had cell toxicity effects (data not shown). Out of 16 potential candidates, a fraction from strain D051 was selected for compound isolation. Using the bioassay as a guide, SM compound **1** was isolated as described above based on a number of factors, including potency, chemical complexity, and biomass yield.

**FIG 1:**
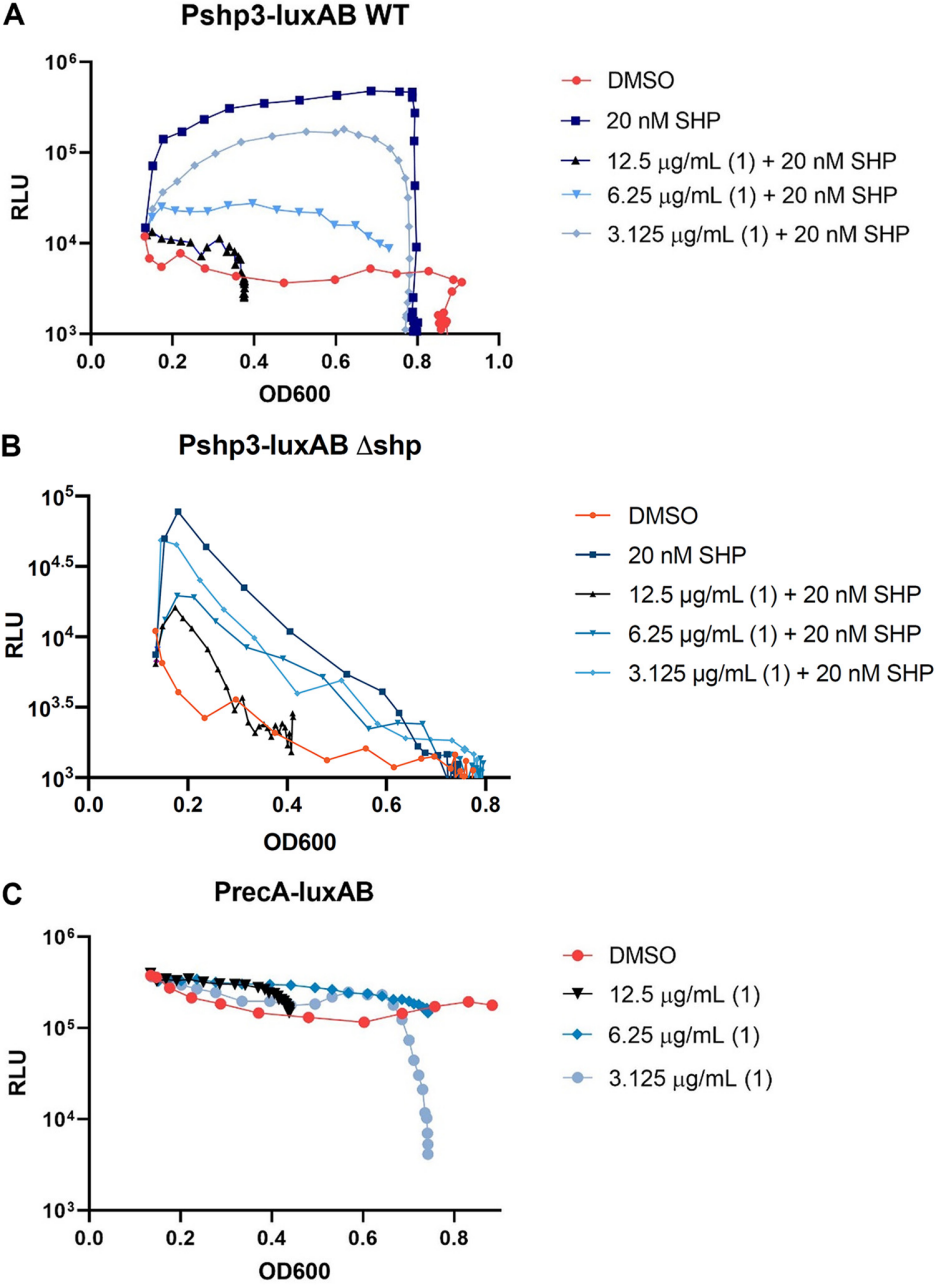
SM 1 is an inhibitor of the Rgg2/3 quorum sensing system in Streptococcus pyogenes. (A) Wild-type (WT) S. pyogenes containing a *P_shp3_-luxAB* transcriptional reporter (JJC 181). (B) S. pyogenes Δ*shp* containing a *P_shp3_-luxAB* transcriptional reporter (BNL206). (C) Wild-type (WT) S. pyogenes containing a *P_recA_-luxAB* transcriptional reporter (JF 02). Cultures were incubated with DMSO (orange) or increasing concentrations of **1** (light blue, 3.125 μg/mL; blue, 6.25 μg/mL; and black, 12.5 μg/mL) followed by 20 nM SHP (dark blue). Graphs shown are representatives of at least three experiments.

To quantify the activity of **1**, we evaluated a range of concentrations (3.125 to 12.5 μg/mL) against the wild-type *P_shp3_-luxAB* (Fig. 1A) or *P_recA_-luxAB* (Fig. 1C) transcriptional reporters and compared them to the dimethyl sulfoxide (DMSO) control. Similar to the original assay, **1** was first added to the cultures, followed by 20 nM SHP. We observed the highest increase in *P_shp3_-luxAB* expression in the presence of SHP alone (Fig. 1A). Expression in the SHP sample increased over time as SHP is produced naturally by the QS system. Titration of **1** starting from 3.125 μg/mL up to 12.5 μg/mL led to a 10-fold decrease in *P_shp3_-luxAB* expression. We observed that 6.25 μg/mL provided the maximal *P_shp3_-luxAB* inhibition compared to the other titration samples and this effect was sustained over time. In comparison, **1** did not affect *P_recA_-luxAB* expression even at its highest concentration used for this assay (Fig. 1C) suggesting that the inhibitory effects of **1** are specific only to the Rgg2/3 signaling pathway and did not affect luciferase or general transcription activities.

Additionally, a consistent inhibition of growth was observed for both reporter strains when **1** was applied at 12.5 μg/mL (Fig. 1). This effect was also observed when performing the same assay in the absence of added SHP (Fig. S2), suggesting that **1** exhibited growth inhibition properties at higher concentrations.

To determine any changes due to positive feedback loops by naturally made SHP, the titration experiment was repeated using a *shp* deletion strain (BNL 206) (13) (Fig. 1B). In this assay, *P_shp3_-luxAB* induction and its continued expression was dependent on exogenously added SHP. The highest increase in *P_shp3_-luxAB* activity was observed when only SHP was provided. Increasing concentrations of **1** in the presence of exogenous SHP showed similar inhibitory effects on *P_shp3_-luxAB* expression. Based on these results, **1** inhibits *P_shp3_-luxAB* expression in a concentration-dependent manner.

### Inhibitory activity of secondary metabolite 1 extends to all Rgg QS systems in S. pyogenes.

**FIG 2:**
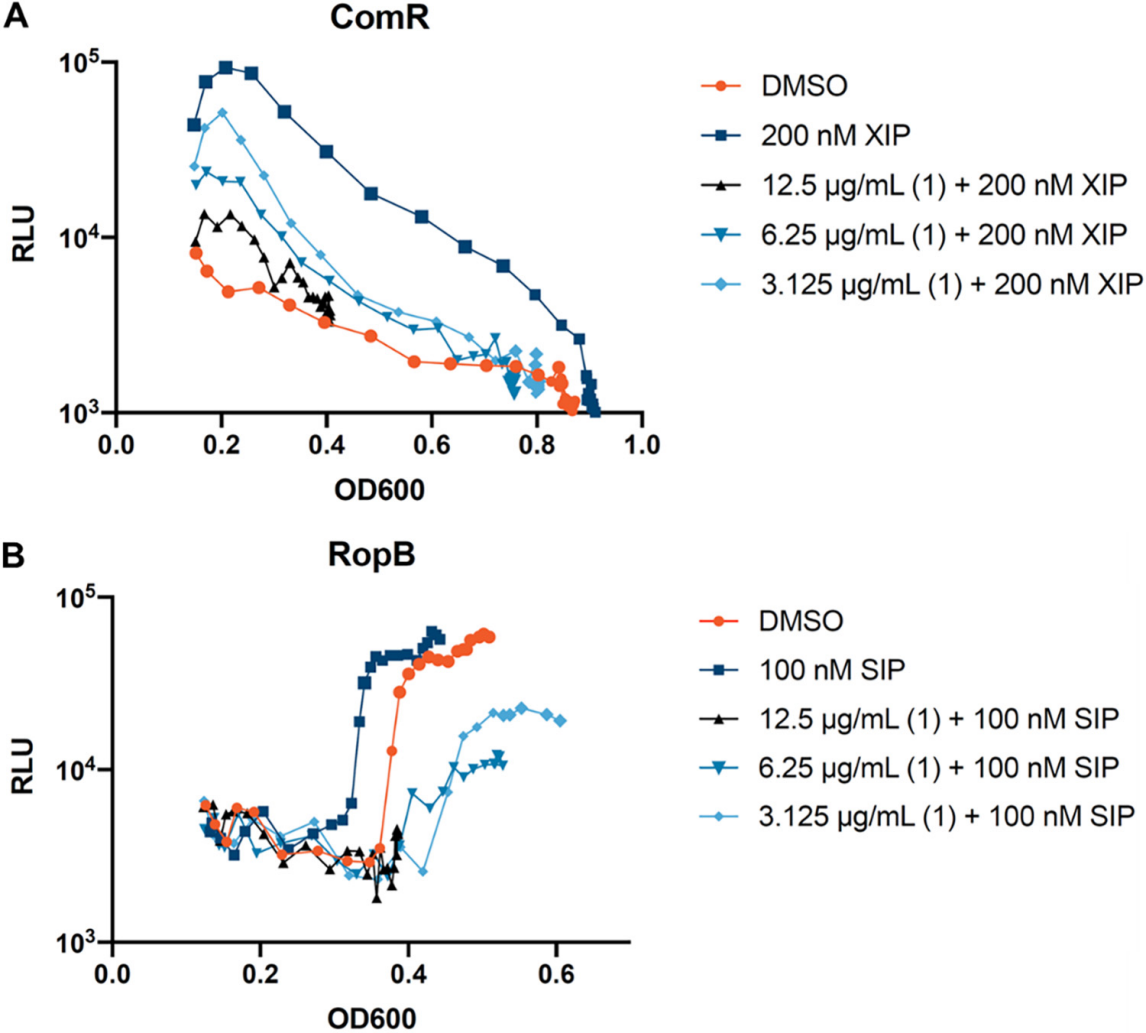
SM 1 inhibits the ComR and RopB quorum sensing system in Streptococcus pyogenes. (A) S. pyogenes *comR* containing a *P_comS_-luxAB* transcriptional reporter (MW 354). (B) S. pyogenes containing a *P_speB_-luxAB* transcriptional reporter (MW 185). Cultures were incubated with DMSO (orange) or increasing concentrations of **1** (light blue, 3.125 μg/mL; blue, 6.25 μg/mL; and black, 12.5 μg/mL) followed by 200 nM XIP or 100 nM SIP (dark blue). Graphs shown are representatives of at least three experiments.

S. pyogenes contains two other Rgg-type QS circuits that are mediated by the pheromone receptors ComR and RopB (8, 9). ComR is known for activating a cryptic natural competence system while RopB leads to virulence factor production. We evaluated **1** against the remaining Rgg systems in S. pyogenes after establishing its inhibition of Rgg2/3 QS. Cultures expressing *P_comS_-luxAB* (for ComR) (MW354) or *P_speB_-luxAB* (for RopB) (MW185) were challenged with a gradient of **1** concentrations followed by the addition of their peptides XIP (200 nM) and SIP (100 nM), respectively (8, 9, 21) (Fig. 2).

Increased *P_comS_-luxAB* expression was observed with the addition of 200 nM XIP. Similar to the Rgg2/3 QS system, a concentration-dependent decrease in *P_comS_-luxAB* expression was observed even in the presence of XIP (Fig. 2A). In the RopB QS system, under SIP-only conditions, we observed induction of the *P_speB_-luxAB* reporter at a lower cell density (Fig. 2B). Over time, the DMSO control increased in *P_speB_-luxAB* expression due to natural production of SIP by the strain. When **1** was added at increasing concentrations, there was a lag in activation and a modest decrease of *P_speB_-luxAB* expression in the presence of SIP. Based on the data collected, **1** can act as a general inhibitor of all Rgg QS systems in S. pyogenes. To the best of our knowledge, this finding represents the first example of a general Rgg QS inhibitor.

### Inhibitory activity of secondary metabolite 1 can extend to other streptococcal species.

Since **1** displayed inhibitory activity against Rgg QS systems present in S. pyogenes, the next step was to determine whether the extract also inhibited Rgg QS systems in other *Streptococci*. S. porcinus (group E Streptococcus) and S. agalactiae (group B Streptococcus [GBS]) were selected as potential candidates, as they contain Rgg-SHP QS circuits similar to that of S. pyogenes (15)*. S. porcinus* (NCTC10999 [pJC254]) and S. agalactiae (A909 [pSAR110]) strains containing a *P_shp_-luxAB* luciferase reporter were tested against increasing concentrations of **1**, followed by 20 nM SHP (Fig. S3) (21, 31, 32).

*P_shp_-luxAB* activity in GBS did not differ in the presence or absence of peptide as cultures can autoinduce (31). In the presence of increasing concentrations of **1**, a modest decrease in *P_shp_-luxAB* induction was observed. However, concentration did not seem to play a role and could be due to the autoinduction effects (Fig. S3A).

In *S. porcinus*, *P_shp_-luxAB* activity occurred only in the presence of exogenous SHP (Fig. S3B) (21). **1** displayed an ability to modulate transcription modestly in *S. porcinus*, with differences observed between concentrations tested (3.125 to 12.5 μg/mL). Thus, inhibition activity of **1** may extend beyond S. pyogenes potentially due to the high level of similarity among signaling components of the Rgg system.

### Secondary metabolite 1 acts as a transcription inhibitor *in vitro*.

**FIG 3:**
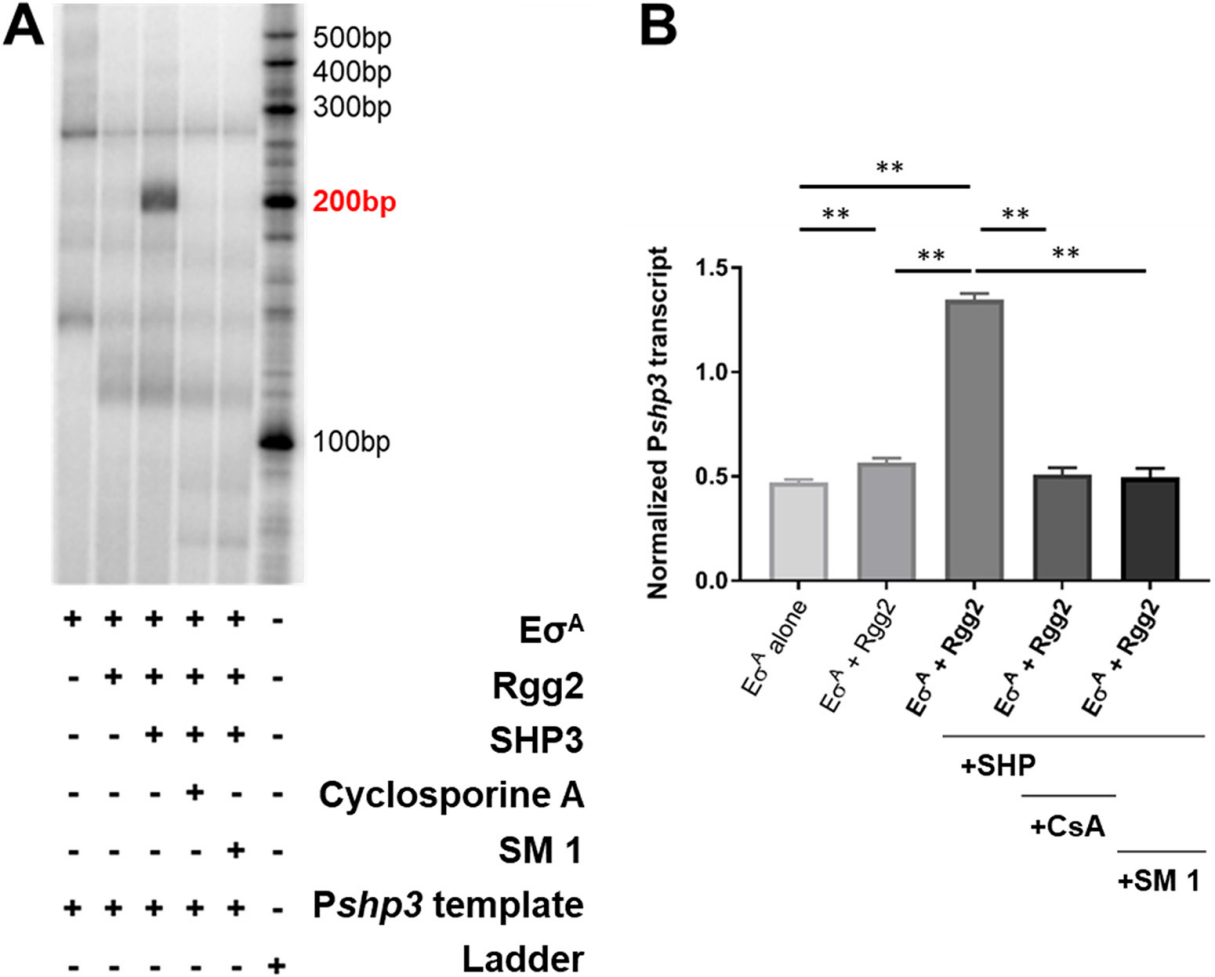
SM **1** inhibits Rgg2-SHP transcription *in vitro*. (A) A representative *in vitro* transcription assay gel. Reaction components are indicated in their corresponding lanes. The template DNA contains the *Pshp3* promoter plus 200 bp from the transcription start site. The product is a 200-bp α^32^P-labeled RNA transcript (indicated in red). (B) *In vitro* transcription results. Target transcript (200 bp band) densitometry was quantified using ImageJ and normalized to the background for each lane. As in the representative gel, reaction 4 includes 100 μg/mL cyclosporine A (CsA), and reaction 5 includes 100 μg/mL of **1**. Data are representative of three independent experiments. Data were analyzed using one-way ANOVA and Tukey’s honestly significant difference test statistical analysis. **, *P* < 0.01.

To assess the ability of **1** to directly interfere with Rgg-dependent transcription, the inhibitor’s ability to block transcription in a cell free *in vitro* system was assessed (33–37). In this system, a double-stranded DNA template of the *P_shp3_* promoter that extends 200 bp downstream of the transcriptional start site was incubated with RNA polymerase holoenzyme (Eσ^A^) alone (negative control), Eσ^A^ + Rgg2, and Eσ^A^ + Rgg2 + SHP (Fig. 3). Test samples included Eσ^A^ + Rgg2 + SHP with 100 μg/mL of a known transcriptional inhibitor, CsA, (21) or **1**. A transcript of 200 nt is visible only in the reaction mixture containing Eσ^A^ + Rgg2 + SHP but not when Eσ^A^ or Eσ^A^ + Rgg2 were provided, demonstrating the pheromone-dependent reaction. The addition of the QS inhibitor CsA or **1** led to no observable transcript, even in the presence of SHP (Fig. 3).

**FIG 4:**
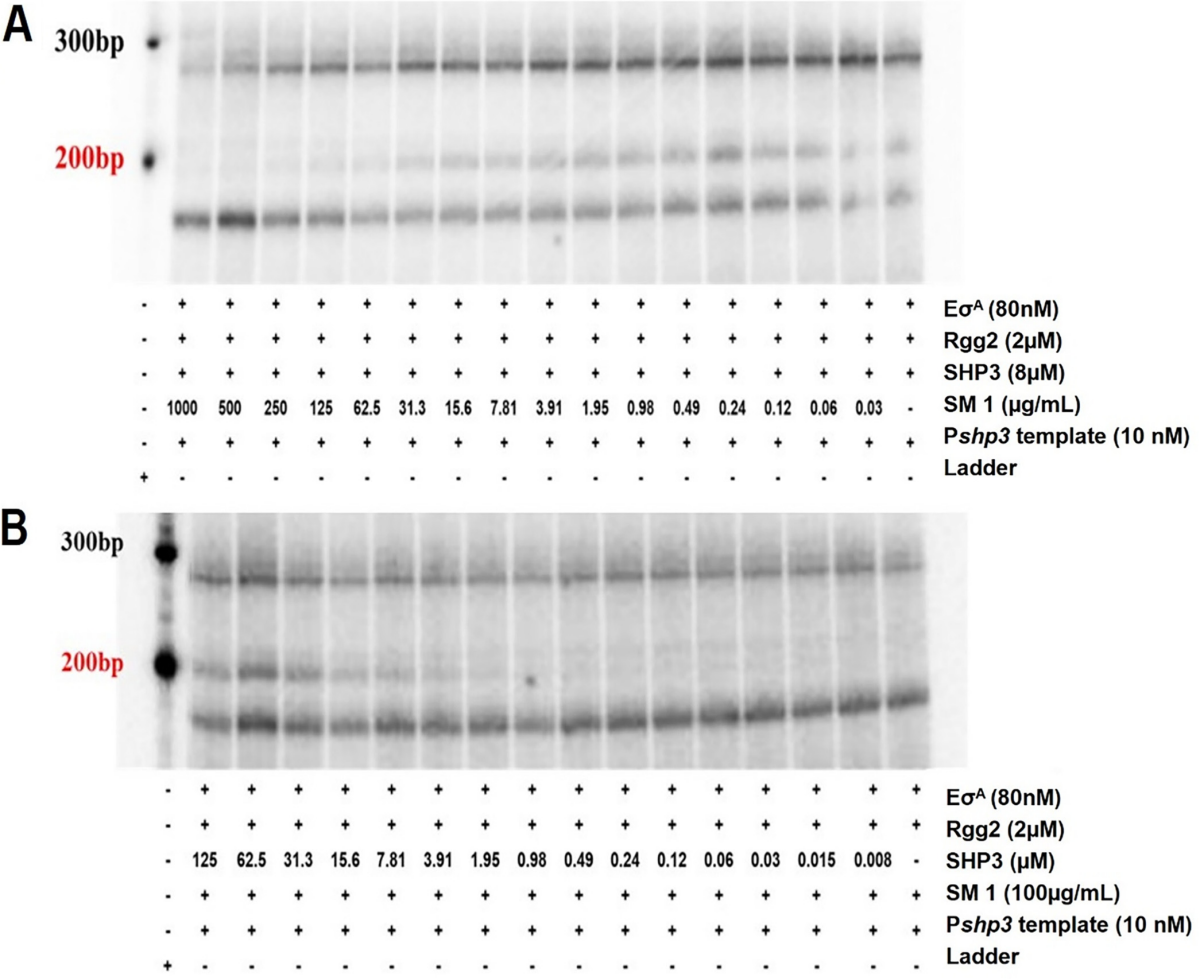
SM **1** competes with the Rgg2-activating peptide SHP *in vitro*. (A) Increasing concentrations of **1** inhibit SHP-dependent transcript activation in a dose-response manner. Reaction components are indicated under corresponding lanes. The template DNA contains the *Pshp3* promoter plus 200 bp from the transcription start site. Target product is indicated in red (SHP3, 8 μM). (B) Increasing concentrations of SHP compete with **1** in a dose-response manner (**1**, 100 μg/mL). Where indicated, reactions in panels A and B include 80 nM holoenzyme, 10 nM dsDNA template, and 2 μM Rgg2. Data are representative of two independent experiments.

To investigate how inhibition of *P_shp3_* transcription occurs based on the *in vitro* data (Fig. 4), a reaction was prepared that contained Eσ^A^ + Rgg2 + SHP (8 μM constant) with increasing concentrations of **1** (0 to 1,000 μg/mL) and presence of *P_shp3_* transcript was determined. As seen in the previous assay, a transcript appeared in the presence of Eσ^A^ + Rgg2 + SHP (Fig. 4A). A transcript was also observed when **1** was added at lower concentrations. However, there was a decrease in transcript starting at 3.9 μg/mL **1** with no observable transcript by 62.5 μg/mL compound 1 (Fig. 4A).

In the next assay, a reverse reaction was prepared containing Eσ^A^ + Rgg2 + **1** (100 μg/mL constant), first with increasing concentrations of SHP (0 to 125 μM) to determine the presence of *P_shp3_* transcript (Fig. 4B). As seen consistently, no *P_shp3_* transcript was observed in the absence of SHP. Similar results were observed when SHP was added at lower concentrations in the presence of **1**. A transcript was only apparent as the SHP concentration continues to increase (3.91 μM), with it being clearly visible at the highest SHP concentrations (31.3 to 125 μM). Our combined data suggest that **1** inhibits *P_shp3_* transcription by competing with the SHP signal *in vitro.*

## DISCUSSION

Quorum-sensing (QS) systems are an important bacterial mechanism used for social responses against environmental cues. They have also been targeted by numerous studies to find alternative therapies to combat bacterial pathogens. We have identified a SM, **1**, produced by Streptomyces tendae D051 with general inhibitory activity against the Rgg QS systems present in the pathogen Streptococcus pyogenes. Through our *in vitro* work, we demonstrate that this metabolite inhibits the Rgg QS regulation via competition with the cognate signaling peptides. Mechanism of action studies, beyond the described activity herein, are under way.

Despite employing a variety of spectroscopic and spectrometric approaches, the full structure of **1** has yet to be elucidated and is ongoing. Herein, we provide a potential hypothesis for the mechanism of action of **1**, based on our work and published literature on Rgg and small molecule interactions. In *in vitro* studies, **1** inhibited transcription in a concentration-dependent manner similar to the cyclic compound CsA (21). In addition, we observed that inhibition activity of **1** was reversible since addition of increasing concentrations of SHP leads to increased transcription *in vitro.* We can hypothesize that **1** is inhibiting SHP binding allosterically by interfering with the binding pocket. This could explain how **1** can maintain QS inhibition in cell cultures even in the presence of exogenously added SHP but not in an *in vitro* system where SHP can be added at higher concentrations.

It is important to note that CsA exhibited inhibitory activity in other streptococcal species that contained Rgg2/3-like proteins but had no effect on S. pyogenes ComR and RopB (21). In comparison, **1** had a modest effect on other streptococcal species and inhibited the Rgg proteins present in S. pyogenes (Fig. 2). One hypothesis is that there might be cell membrane permeability differences in these species that may affect the entry of **1**. An additional hypothesis is that the inhibitory activity of **1** is constricted to the C-terminal conformations created by these Rgg proteins and their peptides specifically in S. pyogenes, but this seems unlikely due to the dissimilarity of the natural ligands among RopB, Rgg2/3, and ComR. Full elucidation of **1** would allow more specific protein-molecule interaction studies.

Herein, we identified a species-specific Rgg QS inhibitor produced by the Gram-positive bacterium Streptomyces tendae D051. **1** provides a new avenue to screen for molecules that can inhibit not only streptococcal species QS pathways but also systems present in other pathogenic bacteria. Future work should focus on understanding the molecular pathways involved in the production of these natural QS inhibitors.

## MATERIALS AND METHODS

### Bacterial growth conditions.

S. pyogenes strains used in this study are shown in Table 1. Cultures were grown in chemically defined media (CDM) supplemented with 1% glucose (6, 8, 38) or C-medium (39) in a 37°C water bath or incubator. To maintain the transcriptional reporter plasmids in S. pyogenes, erythromycin (erm) was used at 1 μg/mL.

**TABLE 1:** Strains used in this study

Strain	Description	Reference
JF02	S. pyogenes *NZ131 P_recA_-luxAB erm^R^*	This study
JCC181	S. pyogenes *NZ131 P_shp3_-luxAB erm^R^*	6
BNL206	S. pyogenes *NZ131 P_shp3_-luxAB erm^R^ shp2GGG shp3GGG*	14
MW354	S. pyogenes *NZ131 comR P_comS_-luxAB erm^R^*	19
MW185	S. pyogenes *NZ131 P_speB_-luxAB erm^R^*	21
A909 (pSAR110)	S. agalactiae *P_shp1520_-luxAB spec^R^*	31
NCTC 10999 (pJC254)	*S. porcinus ATCC43138 P_shp3_-luxAB spec^R^*	21, 32

### Quorum-sensing peptides.

Synthesized Streptococcus pyogenes SHP (short hydrophobic peptide; DIIIIVGG), XIP (SigX inducing peptide; SAVDWWRL), and SIP (SpeB-inducing peptide; MWLLLLFL) used for transcriptional luciferase assays were purchased as dehydrated powder with purity ranging from 30 to 60% (%) and resuspended in DMSO to a 1-mM stock and diluted to required assay concentrations as previously described (NeoPeptide; reference 14). DMSO was used as the negative control for the biological luciferase and growth assays.

### Initial natural products luciferase screen.

A library of 2,500 SM fractions derived from 625 environmental *Actinobacteria* was used in this study. The library was stored in DMSO and diluted in CDM to final test concentrations of 50, 25, 12.5, and 5 μg/mL. Diluted fractions were added to flat bottom, white wall 96-well plates with surfactant-treated lids (14). Plates were then inoculated with 200 μL of exponentially growing S. pyogenes strain JCC181 containing a *P_shp3_-luxAB* luciferase reporter (6). Culture samples were incubated for 30 min before the addition of 200 nM SHP peptide. Luminescence (LUM) and growth (optical density) at 600 nm [OD_600_] measurements were taken after 3 h using a Biotek Synergy 2 microplate reader. Assays were performed in triplicate followed by a validation test.

### Cultivation and extraction of Streptomyces tendae metabolites.

Sequencing of the 16S rRNA gene identified the producing bacterial strain D051 as Streptomyces tendae. Strain D051 was cultivated in 28 × 1-L Fernbach flasks containing high-nutrient media components in artificial seawater (10 g starch, 4 g yeast extract, 2 g peptone, 1 g CaCO_3_, 100 mg KBr, 40 mg FeSO_4_, and 33.3 g Instant Ocean per liter of distilled H_2_O). The cultures were aerated at 160 rpm on Innova 5000 gyratory shaker for 7 days at room temperature. Sterilized Amberlite XAD-16 resin (Sigma-Aldrich; 20 g·L^−1^) was added to the flasks for metabolite absorption and shaken continuously overnight. Cheesecloth was used to filter the resin and cell mass from the culture media. The metabolites were extracted from the resin and cell mass using acetone (2 × 1 L) and then concentrated under reduced pressure. The resulting metabolite concentration was partitioned between water and ethyl acetate (EtOAc). The EtOAc layer was evaporated *in vacuo* to 1.7 g of crude extract. The extract was fractionated via step-gradient silica column chromatography with a solvent system consisting of hexane (Hex), dichloromethane (DCM), and methanol (MeOH) (50:50, Hex:DCM; 0:100, DCM:MeOH; 1:99; 2:98; 5:95; 10:90; 15:85; 20:80; 40:60; 60:40; 80:20) to yield 11 fractions (40).

### Isolation and purification of 1.

A luciferase bioassay guided fractionation was employed to isolate the active metabolite. Fractions 4 to 9 (1.3 g) were active and fractionated using preparative reversed phase (RP) chromatography (10 mL·min^−1^, 40 to 75% aqueous acetonitrile [MeCN] with 0.1% formic acid for 30 min, followed by an isocratic elution of 100% MeCN with 0.1% formic acid for 20 min) to yield 31.2 mg of **1** (retention time, *t*_R_ = 28 min). Preparative high-performance liquid chromatography (HPLC) scale separations were performed using a Waters LC4000 System equipped with a Phenomenex Luna preparative C_18_ column (250 × 21.2 mm, 5 μm) at a flow rate of 10 mL·min^−1^. Semipreparative HPLC scale separations were performed using a Shimadzu system with a Phenomenex Luna semipreparative C_18_ column (250 mm × 10 mm, 5 μm) at a flow rate of 3 mL·min^−1^ (30 to 100% aqueous acetonitrile [MeCN] with 0.1% formic acid for 35 min, followed by an isocratic elution of 100% MeCN with 0.1% formic acid for 20 min, *t*_R_, 22.5 min) (40).

### Analytical experimental detail.

HPLC-UV data were obtained using a Hewlett-Packard series 1100 system controller and pumps with a Model G1315A diode array detector (DAD) (Hewlett-Packard, Palo Alto, CA, USA) equipped with a reversed-phase C_18_ column (Phenomenex Luna, 100 × 4.6 mm, 3 μm) at a flow rate of 0.7 mL·min^−1^.

### Luciferase reporter assay.

S. pyogenes cultures were grown in CDM or C-medium to an OD_600_ of 0.2 to 0.3. Cultures were back-diluted to an OD_600_ of 0.15 and added to a flat-bottom, white-wall 96-well plate with surfactant-treated lids (14). Briefly, cells received treatment or control (DMSO) and were incubated for 30 min. SHP (20 nM), XIP (200 nM), or SIP (100 nM) peptides were added to cultures and placed in microplate reader for the luciferase assay. SM **1** was added at 12.5, 6.25, or 3.125 μg/mL. A kinetic assay was executed that takes LUM and absorbance at OD_600_ measurements every 15 min for 12 h. Relative light units (RLU) are measured as LUM/OD_600_. Max RLU is the time point at which a strain has the highest RLU in comparison to all other test strains or conditions. Assays were performed in triplicate and graphs are representatives of at least three independent experiments.

### *In vitro* transcription.

Runoff *in vitro* transcription assays were performed as described previously (33–37) (ThermoFisher Scientific; AM1308-AM1326). Briefly, 50 nM reconstituted holoenzyme was incubated on ice with 10 nM dsDNA template in a solution of 20 mM Tris-acetate pH 8.0, 20 mM Na-acetate, 10 mM Mg-acetate, 5% glycerol, 14 mM 2-mercaptoethanol, and 0.1 mM EDTA. After the addition of 2 μM purified Rgg2-MBP and indicated concentrations of SHP and/or inhibitor, transcription reactions were started by the addition of 20 μM (each) GTP, ATP, and CTP, and 0.5 μCi/μL ^32^P-UTP. After incubation at 37°C for 10 min, 35 μM cold UTP was added to each reaction and incubated for an additional 20 min at 37°C. The reaction was stopped by the addition of 80% formamide dye and incubation for 2 min at 65°C. Samples were analyzed on a denaturing polyacrylamide gel. The DNA template used was a 261-bp fragment of the *Pshp3* promoter containing 200 bp of transcript and the 61-bp promoter sequence upstream of the +1 transcription start site. The radiolabeled transcript was visualized using a PhosphorImager (Molecular Dynamics).
