# Supplementary material for: A Streptomyces tendae Specialized Metabolite Inhibits Quorum Sensing in Group A Streptococcus
Source: Microbiol Spectr. 2023 Jun 7;11(4):e05279-22. doi: 10.1128/spectrum.05279-22 (PMC10434017; doi:10.1128/spectrum.05279-22)
Supplement: Supplemental file 1 — Supplemental material. Download spectrum.05279-22-s0001.pdf, PDF file, 0.3 MB [file spectrum.05279-22-s0001.pdf]

Supplemental Information

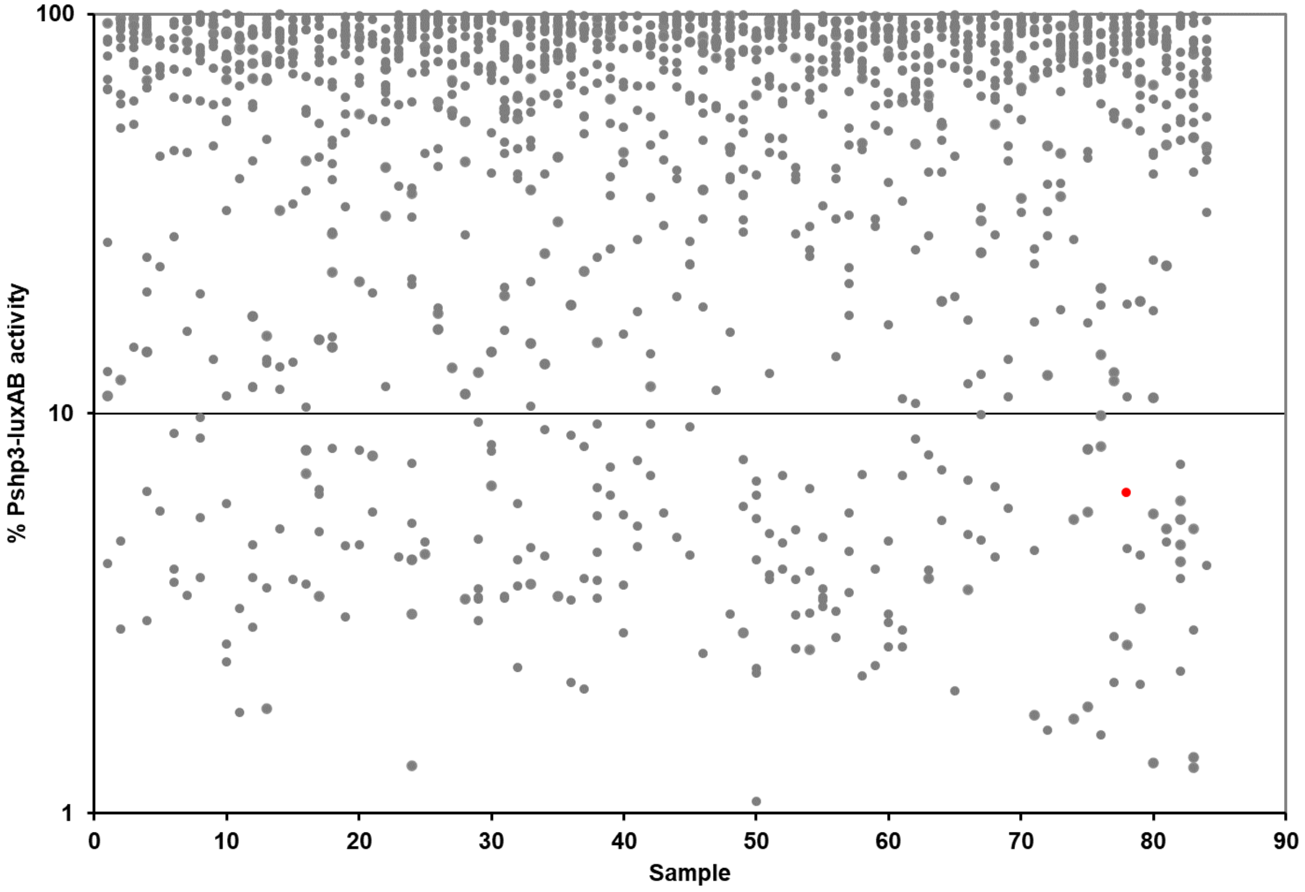

Supplemental Figure 1. Initial actinomycete luciferase screen. Cultures of *Streptococcus pyogenes* containing P_{shp3}-*luxAB* were incubated with 5 µg/mL of actinomycete SM extracts followed by addition of 200 nM SHP. Samples were measured for P_{shp3}-*luxAB* expression and analyzed as % luciferase activity compared to SHP control. Extracts that showed P_{shp3}-*luxAB* expression at 10% or less were considered. Graph shows sample (*x-axis)* vs % P_{shp3}-*luxAB* inhibition *(y-axis).* A total of 30 96-well plates each containing approximately 84 samples are represented in the graph. *Red* dot indicates sample **SM 1**.

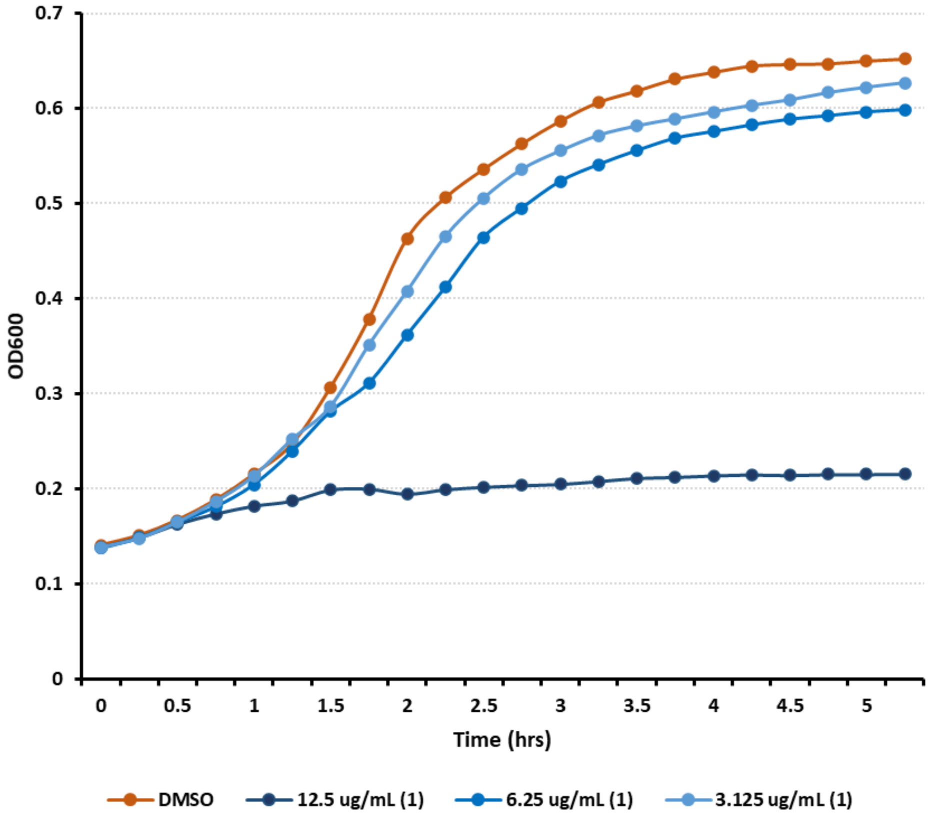

Supplemental Figure 2. Lower **SM 1** concentrations do not affect *S. pyogenes* growth. Cultures of *S. pyogenes* were incubated with increasing concentrations of **1**. Samples were measured for growth (OD600; y-axis) over time (hrs.; x-axis). Orange; DMSO, light blue; 3.125 µg/mL **1**, blue; 6.25 µg/mL **1,** and dark blue; 12.5 µg/mL **1**.

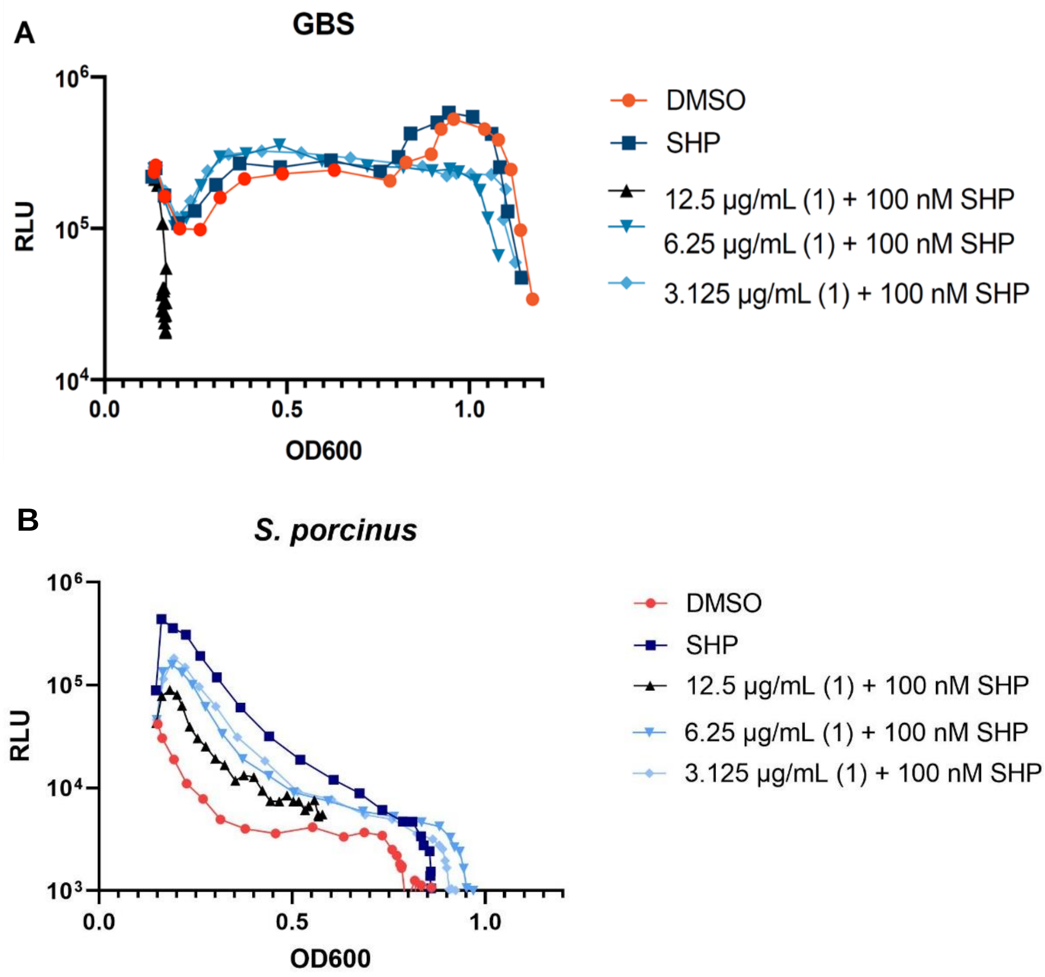

Supplemental Figure 3. SM 1 affect modestly quorum sensing system in other *Streptococcus* species. A) *S. agalactiae* (GBS) containing a P_{shp}-*luxAB* transcriptional reporter (A909(pSAR110)). B) *S. porcinus* containing a P_{shp}-*luxAB* transcriptional reporter (NCTC 10999(pJC254). Cultures were incubated with DMSO (orange) or increasing concentrations of **1** (light blue; 3.125 µg/mL, blue; 6.25 µg/mL, and black; 12.5 µg/mL) followed by 100 nM SHP (dark blue). Graphs shown are representatives of at least three experiments.
